# Retraction: Sudden infant death syndrome: Melatonin, serotonin, and CD34 factor as possible diagnostic markers and prophylactic targets

**DOI:** 10.1371/journal.pone.0294548

**Published:** 2023-11-13

**Authors:** The PLOS ONE Editors

Following the publication of this article [1], concerns were raised regarding Figs 1, 5, and 6 Specifically,

- In Fig 1, there appears to be an overlapping region in the A and B panels despite these panels representing CD34 expression in a control group and infants with Sudden Infant Death Syndrome (SIDS) respectively.
- In Fig 5, there appears to be an overlapping region in the A and B panels despite these panels representing melatonin receptor 1 expression in infants with SIDS and a control group respectively.
- In Fig 6, there appears to be an overlapping region in the B and D panels despite these panels representing melatonin receptor 1 and melatonin receptor 2 expression respectively.

Whilst the underlying data for these panels is provided via a repository of the figures and datasets presented in the published article, the above concerns are additionally present in the underlying data and therefore they do not resolve the concerns. The authors did not provide a response to concerns raised about Figs 1, 5, and 6.

In light of the concerns affecting multiple figure panels that question the integrity and reliability of these data, the *PLOS ONE* Editors retract this article.

All authors either did not respond directly or could not be reached.
